# Comparative Genomic and Functional Characterization of *Pediococcus acidilactici* Isolated from Fermented Cacao with Anti-ESKAPE Activity

**DOI:** 10.3390/ijms27135996

**Published:** 2026-07-03

**Authors:** Pinkanok Suksabay, Yosita Leepromma, Benyapa Prakit, Tansuda Puchong, Joo Shun Tan, Chonticha Romyasamit

**Affiliations:** 1 College of Graduate Studies, Walailak University, Nakhon Si Thammarat 80160, Thailand; pinkanok.su@mail.wu.ac.th (P.S.); tansuda6211@gmail.com (T.P.); 2 Bioprocess Technology Division, School of Industrial Technology, Universiti Sains Malaysia, Gelugor 11800, Pulau Pinang, Malaysia; jooshun@usm.my; 3 Department of Medical Technology, School of Allied Health Sciences, Walailak University, Nakhon Si Thammarat 80160, Thailand; 4 Research Center for Microbiome, Systems Biology, and Medical Innovation, Walailak University, Nakhon Si Thammarat 80160, Thailand; 5 Research Center in Tropical Pathobiology, Walailak University, Nakhon Si Thammarat 80160, Thailand

**Keywords:** postbiotic, fermented cacao, *Pediococcus acidilactici*, ESKAPE pathogens, antimicrobial activity, whole-genome analysis

## Abstract

ESKAPE pathogens have become a major global health challenge. This study aimed to isolate and characterize LAB from fermented cacao and to evaluate their probiotic properties, preliminary antimicrobial activity against ESKAPE pathogens, safety profiles, and functional bioactivities. Each of *P. acidilactici* isolates exhibited antibacterial activity against ESKAPE pathogens, with inhibition zone diameters ranging from 10.00 ± 1.00 mm to 23.00 ± 0.00 mm, depending on the isolate and pathogens tested. CR05 was identified as the most promising probiotic candidate, showing the highest survival at pH 2 (69.65 ± 6.66%), strong tolerance to pancreatin (99.95 ± 0.10%), pepsin (89.11 ± 2.38%), bile salts (98.65 ± 0.33%), and favorable adhesion properties, including auto-aggregation, cell surface hydrophobicity, and adhesion to HT-29 intestinal epithelial cells. The safety assessment indicated a notable susceptibility to gentamicin, tetracycline, and chloramphenicol, with resistance to several other tested antibiotics. and showed no hemolytic activity. Three selected isolates were evaluated for minimum inhibitory concentration (MIC) and minimum bactericidal concentration (MBC) against ESKAPE pathogens, with MIC and MBC values ranging from 12.5 to 25 mg/mL and 25 to >50 mg/mL, respectively. The strongest inhibitory activity was observed against *A. baumannii*, *P. aeruginosa*, and *E. aerogenes*, with MIC values of 12.5 mg/mL, particularly for isolates CR05 and CR06. Whole-genome analysis identified genes related to stress response and gastrointestinal tolerance and predicted the enterolysin A gene. No acquired antimicrobial resistance genes were detected. These findings suggest that *P. acidilactici* isolates from fermented cacao are promising probiotic candidates for further investigation in functional food, probiotic, and postbiotic-related applications.

## 1. Introduction

Antimicrobial resistance (AMR) has emerged as a major public health concern because bacteria have developed resistance to multiple classes of antibiotics [1]. The ESKAPE group of six nosocomial pathogens comprises both Gram-positive and Gram-negative bacteria, including *Enterococcus faecium*, *Staphylococcus aureus*, *Klebsiella pneumoniae*, *Acinetobacter baumannii*, *Pseudomonas aeruginosa*, and *Enterobacter* species, as designated by the World Health Organization (WHO). These pathogens are of particular concern due to their remarkable ability to evade antimicrobial agents and rapidly acquire multidrug resistance. The increasing prevalence of antibiotic-resistant ESKAPE bacteria has created an urgent need for novel therapeutic strategies. Consequently, researchers worldwide are developing alternative therapies to enhance bacterial eradication [2].

Probiotics are living microorganisms that confer health benefits to the host when consumed in proper amounts [3]. Fermentation is a process in which microorganisms catabolize a carbon source to generate energy in the absence of oxidation. The end products of microbial fermentation include alcohols and organic acids such as acetic acid, propionic acid, and lactic acid. Lactic acid bacteria (LAB) are the microorganisms that mainly produce lactic acid as the end product of metabolic activity. [4]. LAB, including the species *Lactobacillus*, *Bifidobacterium*, *Enterococcus*, *Streptococcus*, *Lactococcus*, *and Pediococcus*, are Gram-positive, non-spore-forming, facultatively heterofermentative, catalase-negative microorganisms and are the most frequently used in human applications [5]. They confer health benefits, including antimicrobial activity, through several mechanisms, such as competitive exclusion, adhesion to the intestinal mucosa, immunomodulation, enhancement of epithelial barrier integrity, and production of bacteriocins, hydrogen peroxide, organic acids, and antimicrobial peptides [6]. *Pediococcus acidilactici* is one of the LAB [5]. This strain can produce pediocin, which is an antimicrobial peptide that is applied in food preservation [7]. Recently, a study reported that *P. acidilactici* strains isolated from fermented fish in Thailand exhibit strong probiotic properties, including tolerance to acidic and bile salt conditions, as well as high potential for antimicrobial and antioxidant activity [8]. In the *E. faecium*-colonized mouse model, *P. acidilactici* pediocin reduces *E. faecium* at 3 days postinfection and shows strong activity against vancomycin-resistant *Enterococcus* spp. [9,10]. Biosurfactants produced by *P. acidilactici* show potential as biological agents for preventing or reducing *S. aureus* biofilm formation and suggest their potential use as wound dressing [11] and exhibit strong antibacterial activity against *K. pneumoniae*, *E. coli*, *B. cereus*, *Bacillus subtilis*, *Shigella flexneri*, *P. aeruginosa*, *A. baumanii*, and *Enterobacter* spp. [12,13,14,15]. Although *P. acidilactici* has been widely reported in diverse fermented foods, it has not been previously described in fermented cacao, which represents a potentially valuable source of lactic acid bacteria (LAB) with antimicrobial properties. In contrast to conventional studies that predominantly utilize liquid cell-free supernatants (CFS), the present study employs lyophilization to enhance the stability and potency of bioactive compounds. Therefore, this study aimed to isolate *P. acidilactici* from fermented cacao and evaluate its probiotic potential, focusing on its antimicrobial activity against ESKAPE pathogens using the agar well diffusion method. The antimicrobial efficacy was further assessed by determining the minimum inhibitory concentration (MIC) and minimum bactericidal concentration (MBC). Furthermore, whole-genome sequencing (WGS) was conducted to provide deeper insights into the genetic determinants underlying antimicrobial activity and safety.

## 2. Results

### 2.1. Sample Collection and Isolation of Pediococcus acidilactici

A total of 33 colonies with diverse morphologies were initially recovered from day 5 fermented cacao samples on MRS agar. Based on preliminary antimicrobial screening, 14 isolates showed inhibitory activity against ESKAPE pathogens. Among these, eight isolates were identified as *P. acidilactici* by MALDI-TOF MS and selected for further characterization. The isolates were assigned as CR03, CR04, CR05, CR06, CR07, CR08, CR11, and CR12 (Supplementary Table S1). Whole-genome sequencing analysis further supported that all isolates were identified as *P. acidilactici*. The selected isolates therefore represent culturable LAB candidates recovered under the conditions used in this study.

### 2.2. Preliminary Antimicrobial Activity Against ESKAPE Pathogens

The antimicrobial activity of eight *P. acidilactici* isolates was evaluated using the well-diffusion method. The 8 isolates exhibited inhibitory activity against ESKAPE pathogens (Table 1). The inhibition zone diameters ranged from 10.00 ± 1.00 mm to 23.00 ± 0.00 mm, depending on the isolate and pathogen tested. The results showed *P. aeruginosa* exhibited the largest inhibition zones with all strains, with values ranging from 19.00 ± 0.00 mm to 23.00 ± 0.00 mm. Isolates CR08 (23.00 ± 0.00 mm) demonstrated the largest inhibitory activity against *P. aeruginosa*. Similarly, high inhibition was observed against *A. baumannii*, with zone diameters ranging from 15.33 ± 0.58 mm to 23.00 ± 0.00 mm. The largest inhibition zone was shown in CR11 (23.00 ± 0.00 mm). For *S. aureus*, inhibition zones ranged from 11.33 ± 0.58 mm to 15.33 ± 0.58 mm, with the highest activity observed in CR12 (15.33 ± 0.58 mm). In contrast, *E. faecium* showed moderate susceptibility, with the largest inhibition zone observed for CR08 (14.33 ± 1.15 mm). *K. pneumoniae* exhibited relatively smaller inhibition zones (11.67 ± 0.58 mm to 15.67 ± 0.58 mm). Isolates CR08 and CR12 demonstrated broader antimicrobial spectra against both Gram-positive and Gram-negative bacteria, indicating their potential as candidates with enhanced antimicrobial capacity.

### 2.3. Tolerance to Gastrointestinal Tract Condition

The GIT tolerance properties of eight *P. acidilactici* were evaluated under GIT conditions of low pH (pH 2), pepsin, pancreatin, and bile salts (Table 2). At pH 2, isolates CR05 exhibited the highest survival rate (69.65 ± 6.66%), whereas CR12 showed the lowest tolerance (7.99 ± 3.53%). Under pH 3 conditions, higher survival was observed, with CR08 showing the highest value (87.43 ± 3.05). Most isolates showed high viability in pepsin, with the greatest tolerance observed in CR12 (96.49 ± 1.15%). All isolates exhibited strong tolerance to pancreatin, with survival rates exceeding 91%, and isolate CR05 showed the highest survival (99.95 ± 0.10%). Similarly, all isolates demonstrated high resistance to 0.3% bile salts after 4 h of incubation, with survival rates ranging from 97.12% to 98.65%. Overall, isolate CR05 exhibited greater tolerance under simulated gastrointestinal conditions than the other strains.

### 2.4. Auto-Aggregation Ability

**Figure 1:**
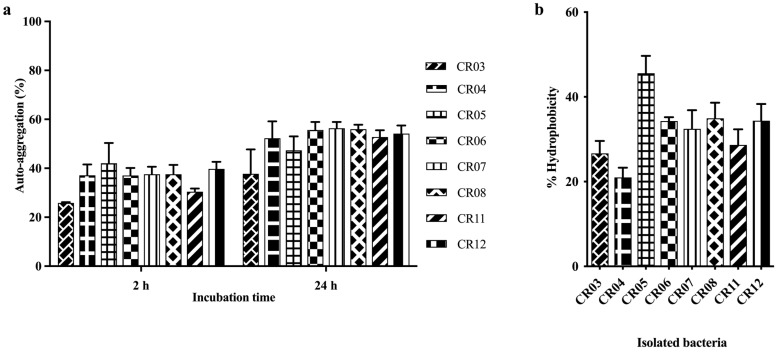
Auto-aggregation and cell surface hydrophobicity of *P. acidilactici*. (**a**) Auto-aggregation (%) of 8 *P. acidilactici* after 2 and 24 h of incubation. (**b**) Cell surface hydrophobicity (%) of *P. acidilactici* in xylene.

The auto-aggregation percentage of the 8 *P. acidilactici* was evaluated. After 2 h, auto-aggregation ranged from 25.85 ± 0.29% (CR03) to 42.05 ± 8.28% (CR05). Following 24 h, all isolates showed increased activity, ranging from 37.76 ± 9.96% to 56.38 ± 2.57%. The highest auto-aggregation percentages at 24 h were observed in CR07 (56.38 ± 2.57%), followed by CR08 (55.97 ± 1.83%). These results are presented in Figure 1a.

### 2.5. Cell Surface Hydrophobicity Ability

The cell surface hydrophobicity of the *P. acidilactici* was evaluated using the microbial adhesion to hydrocarbons (MATH) assay. The results show that all isolates exhibit significant hydrophobicity, with values ranging from 21.01 ± 2.27% (CR04) to 45.56 ± 4.12% (CR05). Based on the classification criteria described in the Methods, all isolates were classified as having low hydrophobicity. Among the tested isolates, CR05 exhibited the highest hydrophobicity (45.56 ± 4.12%). suggesting relatively greater adhesion potential compared with the other isolates (Figure 1b).

### 2.6. Adhesion Ability to Human Intestinal Epithelial Cells

**Figure 2:**
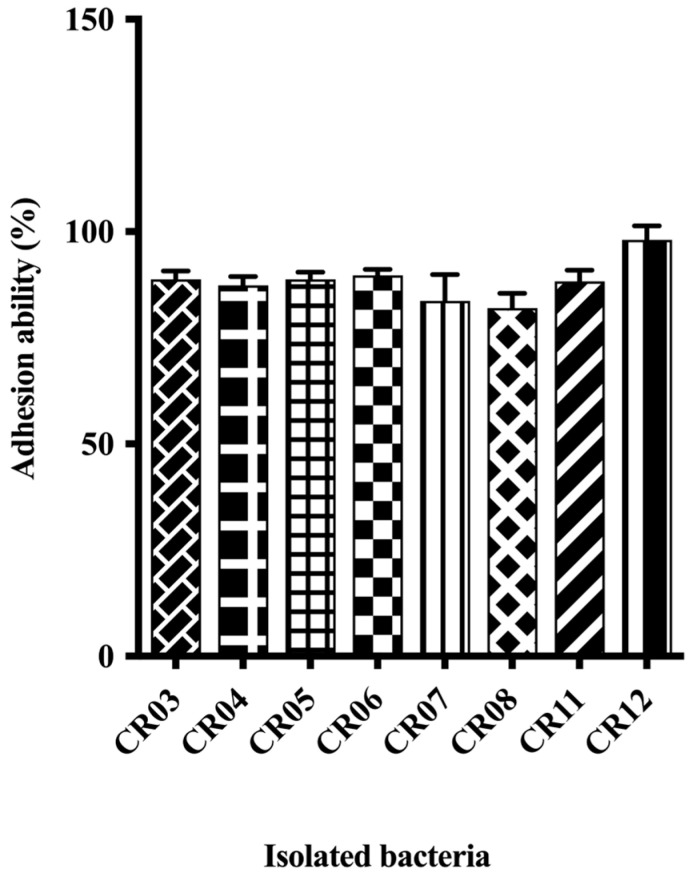
Adhesion ability (%) of *P. acidilactici* to HT-29 cells.

**Figure 3:**
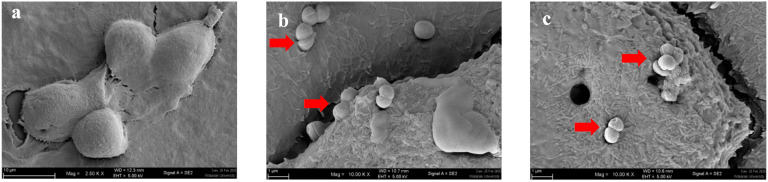
Scanning electron microscope (SEM) analysis of HT-29 cells, where the *P. acidilactici* (CR05) adheres to the surface monolayer of cells. (**a**) Healthy HT-29 cells without any treatment. (**b**,**c**) *P. acidilactici.* The red arrow indicates the attachment of isolated *P. acidilactici* to HT-29 cells.

The adherence ability of the 8 *P. acidilactici* on the HT-29 cell line was determined. The results indicated that all isolates were as strongly adhesive as shown in Figure 2. The number of bacteria adhered to the cells was determined by colony count on MRS agar collected after trypsinization, which enables total enumeration of the bacteria attached to the HT-29 cell line. All isolates demonstrated high adhesion, with adhesion percentages ranging from 82.02 ± 3.52% to 98.03 ± 3.28%. Only one sample from the previous adherence assay was chosen for further analysis by observing under a scanning electron microscope (SEM). HT-29 cells treated with or without selected LAB isolates were observed under SEM, as shown in Figure 3. Untreated HT-29 cells as a control showed healthy cells under ×2500 magnification (Figure 3a). The presence of isolate adhering to the HT-29 cell surface under ×10,000 magnification is shown in Figure 3b and Figure 3c respectively.

### 2.7. Safety Profile Assessment

The safety of the 8 isolates was evaluated using hemolytic and antibiotic susceptibility tests. None of the isolates exhibited hemolytic activity on blood agar plates, indicating γ-hemolysis (non-hemolytic). Additionally, all 8 *P. acidilactici* were susceptible to gentamicin, tetracycline, chloramphenicol, but resistant to ampicillin, vancomycin, erythromycin, clindamycin, kanamycin, and streptomycin (Supplementary Table S2).

### 2.8. MIC and MBC

According to the preliminary antimicrobial assay, isolates CR05, CR06, and CR07 were selected for further determination of minimum inhibitory concentration (MIC) and minimum bactericidal concentration (MBC) against ESKAPE pathogens (Table 3). MIC values ranged from 12.5 to 25 mg/mL. The lowest MIC values (12.5 mg/mL) were observed against *A. baumannii* and *P. aeruginosa*. In contrast, higher MIC values (25 mg/mL) were observed against *E. faecium*, *S. aureus*, and *K. pneumoniae*. Most isolates exhibited MBC values > 50 mg/mL, indicating a predominantly bacteriostatic activity. However, bactericidal activity was observed against *K. pneumoniae* and *E. coli* for isolate CR05 (MBC = 25 mg/mL) and against *K. pneumoniae* for CR07 (MBC = 50 mg/mL). In summary, CR05 demonstrated slightly stronger bactericidal activity than the other isolates.

### 2.9. Characterization of Antimicrobial Substances

The antimicrobial activity of selected LAB isolates was further evaluated after pH neutralization and proteinase K treatment. Under untreated conditions, the results showed inhibitory activity against ESKAPE pathogens. After pH neutralization to pH 7.2, the inhibitory activity was markedly reduced in most tested conditions, suggesting that organic acids played an important role in the observed inhibition. Following proteinase K treatment, the inhibitory activity showed variable responses, with some samples retaining inhibition zones and others showing reduced or undetectable inhibition. However, the inhibition zones after proteinase K treatment were generally weak or less clearly defined. These findings suggest that the antimicrobial activity of the selected LAB isolates was largely associated with acid-mediated effects, while a possible contribution of proteinaceous antimicrobial compounds cannot be excluded and requires further confirmation.

### 2.10. Genome Characteristics, Functional Analysis, Genomic Safety Assessment

**Figure 4:**
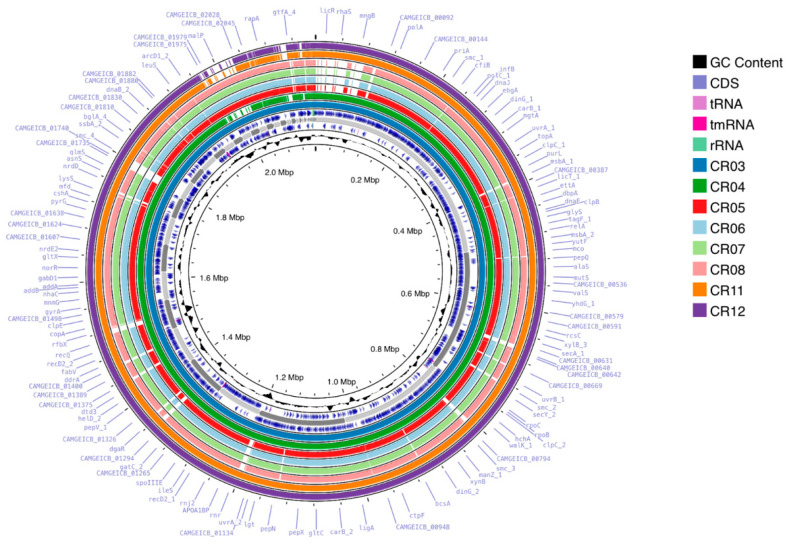
Circular genome map of eight *P. acidilactici* strains generated using Proksee. From outer to inner rings: coding sequences (CDS), tRNA and rRNA genes, followed by comparative genomic rings representing strains CR03–CR12.

The genomic profiles of the eight *P. acidilactici* strains (CR03, CR04, CR05, CR06, CR07, CR08, CR11, and CR12) isolated from fermented cacao were characterized to evaluate their genetic potential. The assembly revealed genome sizes ranging from 2,016,379 bp to 2,154,778 bp, with strain CR03 exhibiting the largest genome, followed by CR08, while CR11 represented the smallest genome. The GC content was highly conserved among all eight strains, remaining stable, ranging between 42.07% and 42.18%. N50 values ranged from 196,543 bp (CR03) to 351,314 bp (CR06, CR07, and CR08). The number of contigs varied from 19 (CR06) to 44 (CR03), where CR06 achieved the highest assembly contiguity (19 contigs), whereas CR03 was the most fragmented (44 contigs). Genome annotation revealed various genetic components, finding that the number of coding sequences (CDS) ranged from 1942 to 2105 genes. The strain with the highest number was CR03 (2105 genes), and the lowest were CR04 and CR12 (1942 genes). All strains contained 3–6 rRNA genes, 52–53 tRNA genes, and one copy of the tmRNA gene (Table 4 and Figure 4). In addition to general genomic characteristics, an analysis was conducted on genes related to survival and specific functions of the eight *P. acidilactici* isolates (Table 5). The results showed that all isolates possessed genes involved in various thermal stress responses and acid tolerance and that all strains possessed a complete set of the F1F0-ATPase operon (atpA–H).

Furthermore, genes related to bile salt tolerance were detected, specifically nagB and pyrG, along with genes involved in osmotic stress response (opuC, opuCD, and opuCC) in all isolates. Analysis using the BAGEL4 platform predicted putative bacteriocin-related gene clusters in all isolates (Supplementary Figure S1). In particular, an enterolysin A-related gene, corresponding to a class III bacteriocin-associated feature, was detected in all isolates. However, this prediction reflects genomic potential rather than confirmed bacteriocin activity. A comprehensive genomic safety assessment was conducted for all eight *P. acidilactici* isolates. Screening with the ResFinder program revealed no acquired antimicrobial resistance genes (ARGs) in the genomes of any strain. This genomic evidence is consistent with the phenotypic antibiotic susceptibility results, which demonstrated that all strains were susceptible to gentamicin, tetracycline, and chloramphenicol. In contrast, the strains exhibited intrinsic resistance to ampicillin, vancomycin, erythromycin, clindamycin, kanamycin, and streptomycin (Supplementary Table S2). Simultaneously, genomic evaluation using the Virulence Factor Database (VFDB) identified no genes associated with bacterial virulence in any of the isolates. These findings reflect that the studied strains lack the essential genetic components related to pathogenicity, host tissue damage, or evasion of the host immune system. Overall, the genomic findings supported some phenotypic observations, particularly stress tolerance and safety-related traits. However, the enterolysin A-related gene was detected in all isolates and therefore could not explain the strain-dependent differences in antimicrobial activity. These differences may be related to metabolite production, gene expression levels, or other regulatory factors rather than gene presence alone.

## 3. Discussion

The ESKAPE pathogens, including *E. faecium*, *S. aureus*, *K. pneumoniae*, *A. baumannii*, *P. aeruginosa*, and *Enterobacter* spp., pose a serious global health threat due to antimicrobial resistance (AMR) [16]. Therefore, the development of novel antimicrobial agents may not be a sustainable long-term solution. An alternative strategy for addressing AMR involves enhancing the host’s natural defenses through probiotics, prebiotics, or synbiotics. By maintaining a balanced microbiota, these therapies can reduce the risk of infection without further promoting AMR [17]. LAB have gained considerable attention as a promising probiotic due to their ability to promote microbiota balance and produce a variety of antimicrobial metabolites [18].

Fermented foods, such as yogurt, cheese, kimchi, and fermented cacao, are widely recognized as the primary niche that supports the activity of LAB [19]. The pulp of Theobroma cacao is rich in fermentable carbohydrates, particularly glucose and fructose, and organic acids like citric acid. These are the essential substrates during the fermentation [20]. In cacao pulp fermentation, yeasts convert glucose into ethanol and participate in pectin degradation, forming aldehydes, organic acids, and esters. Subsequently, LAB utilize glucose, fructose, and citric acid to produce lactic acid, acetic acid, and pyruvate, stabilizing the fermentation environment. Physicochemical changes lead to the accumulation of ethanol, low pH, and high temperature, supporting the survival of microorganisms with enhanced stress conditions [21].

In this study, eight LAB isolates from fermented cacao beans in southern Thailand were identified as *P. acidilactici.* All eight isolates exhibited preliminary antimicrobial activity against ESKAPE pathogens, particularly against *P. aeruginosa* and *A. baumannii*. Because agar diffusion assays are affected by compound diffusion properties, these results should be interpreted as preliminary screening data rather than direct evidence of antimicrobial potency or mechanism. Further CFS characterization showed that pH neutralization markedly reduced inhibitory activity in most tested conditions, suggesting that organic acids played an important role in the observed inhibition. Organic acids may inhibit pathogens by lowering environmental pH, disrupting membrane potential, and interfering with cellular metabolism. Proteinase K treatment produced variable responses, suggesting that proteinaceous antimicrobial compounds may contribute in some conditions but were not confirmed as the main inhibitory factor. These results are consistent with previous studies, indicating that probiotic bacteria isolated from the gut of domestic goats (*Capra hircus*) can inhibit the majority of ESKAPE pathogens, with *A. baumannii* exhibiting the highest susceptibility [3]. Moreover, these isolates exhibited probiotic characteristics, including the ability to tolerate gastrointestinal conditions such as low pH, pepsin, pancreatin, and bile salts, adhesion to the epithelial cell, and safety assessment as defined by FAO/WHO guidelines in 2002 [22]. These data, according to a previous study by Sornsenee et al. [23], showed that *L. paracasei* demonstrated strong gastrointestinal tolerance. Furthermore, it exhibited high stability against pancreatic enzymes at pH 8.0 and pepsin at pH 2, together with high adhesion and hydrophobicity, which support epithelial adhesion.

Whole-genome analysis is widely regarded for probiotic strain assessment, as it enables detailed molecular characterizations associated with important traits such as acid and bile tolerance, metabolite production, strain safety, and antibiotic resistance. In this study, the genome of *P. acidilactici* contains temperature stress-related, acid resistance, bile-salt resistance, and osmotic stress genes. Moreover, WGS-based safety indicated the absence of acquired antimicrobial resistance or virulence-associated genes, with phenotypic findings showing non-hemolytic activity and susceptibility to clinically important antibiotics, such as gentamicin, tetracycline, and chloramphenicol. These findings are consistent with Sornsenee et al. [24]. An enterolysin A-related gene was predicted in all *P. acidilactici* isolates. Enterolysin A is a class III bacteriocin-associated protein with reported bacteriolytic activity, potentially through cell wall degradation. This finding is in accordance with Surachat et al. [25], who reported a bacteriocin-encoding locus containing enterolysin A in the genome of *P. acidilactici* HN9. However, the presence of an enterolysin A-related gene in the present study should be interpreted as bacteriocin-related genomic potential and does not confirm gene expression, bacteriocin production, or direct involvement in the observed antimicrobial activity. Overall, the genomic findings supported some phenotypic observations, particularly stress tolerance and safety-related traits. However, the enterolysin A-related gene was detected in all isolates and therefore could not explain the strain-dependent differences in antimicrobial activity. These differences may be related to metabolite production, gene expression levels, or other regulatory factors rather than gene presence alone.

Together with the pH neutralization results, the observed inhibition appears to be mainly associated with acid-mediated effects, while other LAB-derived metabolites, including possible proteinaceous antimicrobial compounds, may also contribute. Further metabolite profiling, purification of active fractions, and gene expression analysis are required to identify the specific compounds responsible for the observed inhibitory activity and to clarify the functional role of the predicted enterolysin A-related gene.

## 4. Materials and Methods

### 4.1. Bacterial Strains and Culture Conditions

Twelve reference strains of pathogenic microorganisms were used as indicators. These include *Staphylococcus aureus* ATCC 4745, *Pseudomonas aeruginosa* ATCC 15692, *P. aeruginosa* 532610, *P. aeruginosa* 4293-04, *P. aeruginosa* 3285-10, *P. aeruginosa* 479-11, *Klebsiella pneumoniae* ATCC 8216, *Salmonella Typhimurium* DMST 22842, *Salmonella Enteritidis* DMST 15676, *Bacillus cereus* DMST 16007, *Acinetobacter baumannii* ATCC 19606, *Escherichia coli* DMST 12743 and *Enterobacter aerogenes*. These microbial strains were cultivated on Trypticase Soy Agar (HiMedia, Mumbai, India) and incubated at 37 °C for 24 h. under aerobic conditions. Each strain was stored at −80 °C in Tryptic Soy broth (HiMedia, Mumbai, India) supplemented with 30% glycerol until further testing.

### 4.2. Sample Collection and Isolation of LAB

Fermented cacao samples were collected from Srakaew Lung-Lek Cocoa Garden (Thasala District) and Na Luang Sen Sub-district (Thung Song District), Nakhon Si Thammarat, Thailand. Samples were collected on day 5 of fermentation, and one fermented cacao sample was obtained from each source. Samples were transferred at room temperature and processed immediately upon arrival at the laboratory. Briefly, 1 mL of each sample was inoculated into 9 mL of de Man, Rogosa, and Sharpe (MRS) broth (HiMedia, Mumbai, India) and incubated at 37 °C for 24 h. After incubation, 1 mL of the culture was spread onto de Man, Rogosa, and Sharpe (MRS) agar (HiMedia, Mumbai, India) and incubated for an additional 24 h. Colonies exhibiting different morphologies, particularly in color and size, were selected, subcultured onto fresh MRS agar plates, and incubated for 24 h. In total, 33 colonies with different morphologies were obtained from MRS agar. Pure colonies were preserved in MRS broth containing 30% (*v*/*v*) glycerol at −80 °C until use.

### 4.3. Screening of Fermented Cacao Isolates Against ESKAPE Pathogens

The agar well diffusion assay was used to determine the antimicrobial activity of the LAB isolates described by Romyasamit et al. [26], with a few modifications. Pathogenic bacteria were suspended in normal saline solution (NSS) to a density equivalent to 0.5 McFarland standard and evenly spread on Mueller-Hinton agar (MHA) plates (HiMedia, Mumbai, India). An overnight LAB culture was used for testing. Six wells, each 6 mm in diameter, were drilled into the agar plates, and 50 µL of 1 × 10^8^ CFU/mL of each isolated LAB culture was added to each well. Then, it was incubated at 37 °C for 18 h. After incubation, the inhibition zones were measured to assess the antimicrobial activity of the LAB isolates. Based on this preliminary antimicrobial screening, 14 isolates showing inhibitory activity against the pathogen were retained for further identification and characterization.

### 4.4. LAB Identification

Each colony was screened for Gram staining, microscopic examination, and catalase activity according to Bergey’s manual [27]. Selected LAB isolates, which are Gram-positive, catalase-negative colonies and against ESKAPE pathogens, were further characterized and confirmed by matrix-assisted laser desorption ionization-time of flight (MALDI-TOF) mass spectrometry (Bruker Daltonics, Karlsruhe, Germany) as per the manufacturer’s instructions. In brief, 3–5 colony isolates were transferred into 300 µL sterile water, then resuspended in 900 µL of ethanol and centrifuged. The pellets were suspended with formic acid and incubated for 30 min. Add 100% of acetonitrile, and the supernatant was collected. Alpha-cyano-4-hydroxycinamic acid (HCCA) matrix solution (10 mg HCCA in 1 mL of OS [500 µL of 5% trifluoroacetic acid and 500 µL of 100% acetonitrile]) was added to the supernatant, and 2 µL of the mixture was spotted on the MALDI plate. The MALDI plate was prepared with double measurements, adhering to quality control standards. MALDI-TOF MS was performed using the Bruker Ultraflex III TOF/TOF system in linear positive mode within a mass range of 2–20 kDa. Spectral data were analyzed using BioTyper 2.0 software for precise identification. Among the 14 isolates with preliminary antimicrobial activity, eight isolates were identified as *Pediococcus acidilactici* and selected for subsequent probiotic characterization, antimicrobial activity assessment, and whole-genome analysis.

### 4.5. Characterization of Probiotic Properties

#### 4.5.1. Acid Tolerance

The acid tolerance of selected LAB isolates was evaluated according to the modified method of Zommara et al. [28]. Selected LAB isolates were cultured on MRS agar and incubated anaerobically at 37 °C for 48 h. After incubation, the colonies were collected and suspended in sterile saline to a 0.5 McFarland standard (1 × 10^8^ CFU/mL). Each suspension was then inoculated into MRS broth adjusted to pH 2, 3, and 6 using 1 N Hydrochloric acid (HCl). Aliquots were serially diluted in sterile saline and drop-plated (10 µL) onto MRS agar. And then incubated at 37 °C for 0 and 3 h. After incubation, viable colonies were counted, and the percentage survival was calculated as:

% Survival Rate = (CFU/mL at 3 h ÷ CFU/mL at 0 h) × 100

#### 4.5.2. Tolerance to Pepsin and Pancreatin

Tolerance to pepsin and pancreatin digestion was determined by Guney et al. [29] with minor modifications. Selected LAB isolates were cultured on MRS agar and incubated anaerobically at 37 °C for 48 h. After incubation, the colonies were collected and suspended in sterile saline to a 0.5 McFarland standard (1 × 10^8^ CFU/mL). Pepsin solution was prepared by adding pepsin (Sigma-Aldrich, St. Louis, MO, USA) to MRS broth at a concentration of 3 g/L. The pH was adjusted to 2.0 using 1 N HCl. Pancreatin solution was prepared by adding pancreatin (Sigma-Aldrich, St. Louis, MO, USA) to MRS broth at a concentration of 1 g/L. The pH was adjusted to 8.0 with 0.1 M NaOH. Each suspension was then inoculated into MRS broth containing pepsin or pancreatin. Aliquots were serially diluted in sterile saline and drop-plated (10 µL) onto MRS agar. Incubated at 37 °C for 0 and 3 h. After incubation, viable colonies were counted, and the percentage survival was calculated as:

% Survival Rate = (CFU/mL at 3 h ÷ CFU/mL at 0 h) × 100

#### 4.5.3. Bile Salt Tolerance

The bile salt tolerance of selected LAB isolates was evaluated according to the modified method of Bentahar et al. [30]. Selected LAB isolates were cultured on MRS agar and incubated anaerobically at 37 °C for 48 h. After incubation, the colonies were collected and suspended in sterile saline to a 0.5 McFarland standard (1 × 10^8^ CFU/mL). Each suspension was then inoculated into MRS broth adjusted to 0.3% (*w*/*v*) bile salt (Sigma-Aldrich, St. Louis, MO, USA) as well as control solutions (without bile salts). The mixtures were incubated at 37 °C for 3 h. After incubation, viable colonies were counted, and the percentage survival was calculated as:

% Survival Rate = (CFU/mL at 3 h ÷ CFU/mL at 0 h) × 100

#### 4.5.4. Auto-Aggregation Assay

The auto-aggregation ability of the selected LAB isolates was assessed using the method described by Bentahar et al. [30]. with slight modifications. Selected LAB isolates were cultured on MRS agar and incubated anaerobically at 37 °C for 48 h. After incubation, the colonies were collected and suspended in sterile saline to a 0.5 McFarland standard (1 × 10^8^ CFU/mL). The suspensions were mixed thoroughly with a vortex mixera nd incubated at 37 °C, and the upper supernatant was measured at 600 nm at 0, 2, 4, and 24 h. The auto-aggregation percentage was determined using the following formula:

Auto-aggregation (%) = [1 − (A_time_∕A_0_) × 100]

A_time_ is the absorbance at a particular time; A_0_ is the absorbance at time 0.

#### 4.5.5. Cell Surface Hydrophobicity Assay

The cell surface hydrophobicity was determined using xylene according to the method described by Kumari et al. [31] with slight modifications. Selected LAB isolates were cultured on MRS agar and incubated anaerobically at 37 °C for 48 h. After incubation, the colonies were collected and suspended in sterile saline to a 0.5 McFarland standard (1 × 10^8^ CFU/mL). The suspensions (1.5 mL) were mixed with 0.5 mL of xylene, incubated at 37 °C, and vortexed for 30 min. For phase separation (an organic phase and an aqueous phase). The aqueous phase was measured at 600 nm. The percentage cell surface hydrophobicity (H%) was calculated using the formula:

Hydrophobicity (%) = [(1 − A_1_)/A_0_] × 100

A_0_: Initial optical density of the cell suspension (initial cell suspension).

A_1_: Optical density of the aqueous cell suspension after phase separation.

H% > 70%: Bacteria exhibit high hydrophobicity.

H% between 50 and 70%: Bacteria exhibit moderate hydrophobicity.

H% < 50%: Bacteria exhibit low hydrophobicity.

#### 4.5.6. Adhesion to Human Intestinal Epithelial Cells

The adhesion of selected LAB isolates to human intestinal epithelial HT-29 cells was measured following the method modified by Alameri et al. [32], with slight modifications. HT-29 cells were cultured in Dulbecco’s modified Eagle’s (DMEM; Thermo Fisher Scientific, Waltham, MA, USA) supplemented with 10% fetal bovine serum and a mixture of antibiotics (50 μg/mL streptomycin-penicillin, 50 μg/mL gentamicin, and 1.25 μg/mL amphotericin B (Thermo Fisher Scientific, Waltham, MA, USA). HT-29 cells were seeded into 24-well plates at 1 mL per well and incubated at 37 °C in a 5% CO_2_ atmosphere until approximately 80% confluence. The culture medium was then removed, and each well was inoculated with 1 mL of selected LAB isolate suspension (1 × 10^8^ CFU/mL). The plates were incubated for 2 h at 37 °C in a 5% CO_2_ atmosphere. After incubation for 2 h, non-adherent selected LAB isolate cells were removed by washing the wells four times with 1 × PBS (pH 7.2). Then, 100 µL of 2.5% (*w*/*v*) trypsin was added to each well, and the plates were incubated at 37 °C in a 5% CO_2_ atmosphere for 5 min to detach the cells. Subsequently, 900 µL of DMEM was added, and the cell suspensions were serially diluted and drop plated (10 µL) onto MRS agar. Adhesion ability (%) was calculated with the following formula:

% Adhesion Ability = (V_1_ × 100)/V_0_

V_1_: The number of adherent bacterial colonies (CFU/mL).

V_0_: The initial concentration of bacterial colonies introduced (CFU/mL).

#### 4.5.7. Scanning Electron Microscopy (SEM) of Adhered Isolated Lactobacillus Toward Human Intestinal Epithelial Cells

The ability of selected LAB isolates to adhere to HT-29 cells was determined using a method described by Sornsenee et al. [33] with a few modifications. HT-29 cells with selected LAB isolates were fixed with 2.5% (*v*/*v*) glutaraldehyde (Sigma-Aldrich, St. Louis, MO, USA) in 0.1 M phosphate buffer for overnight at 4 °C. Then, the cells were dehydrated through a graded ethanol series (20%, 40%, 60%, and 80% *v*/*v*) for 15 min each session, followed by two final dehydrations in 100% ethanol (Fisher Scientific, Fairlawn, NJ, USA) for 15 min each. Subsequently, the specimens were dried using a critical-point dryer (CPD) and observed under a field-emission scanning electron microscope (FE-SEM) (Merlin Compact, Zeiss, Oberkochen, Germany) equipped with energy-dispersive X-ray spectroscopy (EDX; Aztec, Oxford, UK) and electron backscatter diffraction (EBSD; Nordlys Max, Oxford, UK) for morphological examination.

### 4.6. Safety Assessment

#### 4.6.1. Hemolytic Test

The hemolytic activity of selected LAB isolates was assessed following the method described by Zhang et al. [34] to evaluate their safety for potential probiotic application. All selected LAB isolates were streaked onto blood agar plates (HiMedia, Mumbai, India) containing 5% (*w*/*v*) sheep blood, and the plates were incubated at 37 °C for 48 h. This incubation period ensured that even slow-growing strains had sufficient time to proliferate and express potential hemolytic activity. After incubation, the plates were examined for three types of hemolysis: β-hemolysis, α-hemolysis, and γ-hemolysis (non-hemolytic).

#### 4.6.2. Susceptibility to Antibiotics

The antibiotic susceptibility of selected LAB isolates was determined using the antibiotic disk diffusion method as a preliminary screening on MRS agar plates. Selected LAB isolates were streaked onto MRS agar plates and incubated at 37 °C for 48 h. After incubation, the colonies were adjusted to a 0.5 McFarland standard in normal saline solution (NSS). The standardized bacterial suspension was then evenly inoculated onto MRS agar plates using a sterile swab. Antibiotic disks were placed on the inoculated agar surface, and the plates were incubated at 37 °C for 48 h under anaerobic conditions. Antibiotics selected for testing were based on the guidelines from the European Food Safety Authority (EFSA) [35] and included ampicillin (10 μg), vancomycin (30 μg), gentamicin (10 μg), erythromycin (15 μg), clindamycin (2 μg), tetracycline (30 μg), kanamycin (30 μg), chloramphenicol (30 μg), and streptomycin (10 μg) (Oxoid, Hampshire, UK). After incubation, the diameters of the inhibition zones were measured in millimeters and interpreted with reference to CLSI 2025 criteria [36], where applicable. However, as standardized clinical breakpoints for food-associated lactic acid bacteria are limited, the results were interpreted as preliminary susceptibility profiles rather than definitive clinical resistance classifications.

### 4.7. Characterization of Antimicrobial Activity

#### 4.7.1. Determination of Minimum Inhibitory Concentration (MIC) and Minimum Bactericidal Concentration (MBC)

The MIC and MBC of the cell-free supernatants (CFSs) of selected LAB isolates against ESKAPE pathogens were evaluated according to the methods described in the Clinical and Laboratory Standards [36]. Selected LAB isolates were serially diluted in a 96-well microtiter plate to final concentrations ranging from 50 to 0.195 mg/mL in Muller-Hinton Broth (MHB), with 50 µL per well. Subsequently, ESKAPE pathogens (7.5 × 10^5^ CFU/mL) were added to each well in 50 µL. The plates were incubated at 37 °C for 18 h. Tetracycline (TE) and MHB were used as positive and negative controls, respectively. The results were evaluated by observing the turbidity or clarity of the wells. The MBC values were determined by dropping from wells with MIC values indicating bacterial growth inhibition onto Tryptic Soy Agar (TSA) plates to assess bacterial viability.

#### 4.7.2. Characterization of Antimicrobial Substances by pH Neutralization and Proteinase K Treatment

The antimicrobial substances produced by the selected LAB isolates were evaluated according to [33], with slight modifications. Briefly, overnight cultures of selected LAB isolates were centrifuged at 4000 rpm for 10 min, and the supernatants were collected as CFS. Three CFS conditions were prepared: untreated CFS, pH-neutralized CFS, and proteinase K-treated CFS. For pH neutralization, the CFS was adjusted to pH 7.2 using sterile 1 N NaOH. For proteolytic enzyme treatment, the CFS was treated with proteinase K at a final concentration of 1 mg/mL and incubated at 30 °C for 120 min, followed by heating at 80 °C for 10 min to inactivate the enzyme. All treated and untreated CFSs were filtered through 0.2 µm membrane filters. The antimicrobial activity of each CFS condition was evaluated using the agar well diffusion assay. Antimicrobial activity was assessed by agar well diffusion on MHA, and inhibition zones were measured after incubation at 37 °C for 24 h.

### 4.8. DNA Extraction and Genomic Analysis

Whole-genome sequencing and bioinformatic analysis. Genomic DNA of *P. acidilactici* strains was extracted using the DNeasy extraction kit (QIAGEN, Hilden, Germany). DNA quality and purity were assessed based on the A260/A280 absorbance ratio. Whole-genome sequencing and subsequent bioinformatic analyses were performed as previously described by Sornsenee et al. [37]. The raw sequencing data were assembled using SPAdes v4.1.0 [38], and assembly quality metrics, including genome size, GC content, and N50, were assessed using QUAST [39,40]. Genome annotation was performed using Prokka v1.14.6. to identify coding sequences (CDSs), tRNAs, and rRNAs [41]. Genes associated with probiotic characteristics, including temperature, acid, bile salt, and osmotic stress tolerance, were identified from annotation results. Bacteriocin gene clusters were predicted using BAGEL4 [42]. Antibiotic resistance genes and virulence factors were identified using ResFinder v4.7.2 [43] and VirulenceFinder 2.0 [44], respectively. Comparative genomic analysis was performed using Proksee 1.0.0a6 [45] for BLASTn v2.16.0 [46]-based comparisons. Circular genome maps were constructed to visualize genomic features and sequence similarity among isolates.

### 4.9. Statistical Analysis

All experiments were performed in triplicate. The data were analyzed using one-way analysis of variance (ANOVA) in GraphPad Prism 10. Differences among means were tested; *p*-values < 0.05 were considered statistically significant.

## 5. Conclusions

The present study demonstrates that fermented cacao represents a potential source of lactic acid bacteria with probiotic and antimicrobial properties. *P. acidilactici* isolates obtained from fermented cacao exhibited preliminary antimicrobial activity against ESKAPE pathogens, while genomic analysis further supported their safety and functional potential through the absence of acquired antimicrobial resistance genes and virulence-associated genes. In addition, an enterolysin A-related gene was predicted in all isolates; however, this finding should be interpreted as genomic potential rather than confirmed bacteriocin activity. These findings highlight the potential of these isolates as candidates for further development in functional food applications and for further investigation as alternative approaches related to antimicrobial-resistant pathogens. In addition, the application of these isolates or their metabolites in the biosynthesis of silver nanoparticles may be explored to further enhance antimicrobial activity.

## Figures and Tables

**Table 1:** Inhibition zone diameters (mm) of *Pediococcus acidilactici* isolates from fermented cacao against ESKAPE pathogens using the agar well diffusion assay.

Bacteria	CR03	CR04	CR05	CR06	CR07	CR08	CR11	CR12
*E*. *faecium* ATCC 700221	13.33 ± 0.58	13.00 ± 0.00	14.33 ± 0.58	12.33 ± 0.58	11.67 ± 0.58	14.33 ± 1.15	14.00 ± 1.73	14.33 ± 0.58
*S. aureus* ATCC 4745	13.33 ± 0.58	12.33 ± 0.58	11.33 ± 0.58	11.33 ± 0.58	11.67 ± 0.58	15.00 ± 0.00	14.33 ± 0.58	15.33 ± 0.58
*K. pneumoniae* ATCC 8216	15.33 ± 0.58	15.67 ± 0.58	13.67 ± 0.58	13.67 ± 0.58	11.67 ± 0.58	15.00 ± 1.00	14.00 ± 0.00	13.00 ± 0.00
*A. baumannii* ATCC 19606	19.33 ± 0.58	20.33 ± 0.58	20.33 ± 0.58	20.33 ± 0.58	15.33 ± 0.58	17.00 ± 1.73	23.00 ± 0.00	22.00 ± 0.00
*P. aeruginosa* ATCC 15692	21.67 ± 0.58	21.33 ± 0.58	22.33 ± 0.58	22.33 ± 0.58	20.33 ± 0.58	23.00 ± 0.00	19.00 ± 0.00	22.00 ± 0.00
*E. aerogenes*	14.67 ± 0.58	10.00 ± 1.00	19.33 ± 0.58	15.33 ± 1.15	10.67 ± 0.58	10.67 ± 0.58	13.67 ± 0.58	15.33 ± 0.58

Data are expressed as mean ± standard deviation (SD) of three independent experiments.

**Table 2:** Survival rate (%) of *Pediococcus acidilactici* in the GIT condition.

Isolates	pH 2	pH 3	Pepsin	Pancretin	0.3% Bile Salt
CR03	42.12 ± 1.43	45.55 ± 7.34	NG	99.28 ± 1.22	98.01 ± 0.55
CR04	30.18 ± 2.56	80.70 ± 8.03	89.03 ± 2.64	99.43 ± 0.59	98.49 ± 0.32
CR05	69.65 ± 6.66	66.62 ± 1.59	89.11 ± 2.38	99.95 ± 0.10	98.65 ± 0.33
CR06	57.95 ± 7.61	82.69 ± 10.22	85.76 ± 0.50	99.70 ± 0.14	97.12 ± 1.11
CR07	57.01 ± 1.39	80.89 ± 0.32	96.27 ± 0.80	98.38 ± 0.22	98.37 ± 0.55
CR08	18.48 ± 3.83	87.43 ± 3.05	81.99 ± 1.38	99.74 ± 0.27	97.63 ± 0.77
CR11	14.17 ± 2.20	28.73 ± 8.13	93.13 ± 1.32	91.73 ± 0.50	97.90 ± 1.07
CR12	7.99 ± 3.53	7.29 ± 4.18	96.49 ± 1.15	99.88 ± 0.20	97.98 ± 0.28

NG, no growth. Data are expressed as mean ± standard deviation (SD) of three inde-pendent experiments.

**Table 3:** Minimum inhibitory concentration (MIC) and minimum bactericidal concentration (MBC) of *Pediococcus acidilactici* against ESKAPE pathogens.

Bacteria Stain	CR05		CR06		CR07	
	MIC	MBC	MIC	MBC	MIC	MBC
*E. faecium* ATCC 700221	25	>50	25	>50	25	>50
*S. aureus* ATCC 4745	25	>50	25	>50	25	>50
*K. pneumoniae* ATCC 8216	25	25	25	>50	25	50
*A. baumannii* ATCC 19606	12.5	>50	12.5	>50	12.5	>50
*P. aeruginosa* ATCC 15692	12.5	>50	12.5	>50	12.5	>50
*E. aerogenes*	12.5	>50	12.5	>50	25	>50
*E. coli* DMST 12743	25	25	25	25	12.5	>50

**Table 4:** Genome features of *Pediococcus acidilactici* strains.

Feature	CR03	CR04	CR05	CR06	CR07	CR08	CR11	CR12
Total length	2,154,778	2,016,767	2,032,184	2,031,513	2,031,833	2,044,570	2,016,379	2,016,495
GC (%)	42.07	42.18	42.13	42.13	42.13	42.10	42.18	42.18
N50	196,543	274,725	351,304	351,314	351,314	351,314	274,725	274,725
L50	4	3	3	3	3	3	3	3
Contigs	44	24	21	19	20	21	24	25
CDS	2105	1942	1963	1963	1963	1977	1943	1942
rRNA	4	5	6	5	5	3	4	4
tRNA	52	53	53	53	53	52	52	52
tmRNA	1	1	1	1	1	1	1	1

**Table 5:** Genes associated with probiotic functions of *Pediococcus acidilactici* strains.

Function	Gene	CR03	CR04	CR05	CR06	CR07	CR08	CR11	CR12
**Temperature stress**	*dnaK*	+	+	+	+	+	+	+	+
	*dnaJ*	+	+	+	+	+	+	+	+
	*grpE*	+	+	+	+	+	+	+	+
	*hrcA*	+	+	+	+	+	+	+	+
	*clcP*	−	−	−	−	−	−	−	−
	*cspA*	−	−	−	−	−	−	−	−
	*groS*	+	+	+	+	+	+	+	+
	*groL*	+	+	+	+	+	+	+	+
**Acid tolerance**	*atpA*	+	+	+	+	+	+	+	+
	*atpB*	+	+	+	+	+	+	+	+
	*atpC*	+	+	+	+	+	+	+	+
	*atpD*	+	+	+	+	+	+	+	+
	*atpE*	+	+	+	+	+	+	+	+
	*atpF*	+	+	+	+	+	+	+	+
	*atpG*	+	+	+	+	+	+	+	+
	*atpH*	+	+	+	+	+	+	+	+
**Bile salt tolerance**	*acrD*	−	−	−	−	−	−	−	−
	*nagB*	+	+	+	+	+	+	+	+
	*pyrG*	+	+	+	+	+	+	+	+
	*bsh*	−	−	−	−	−	−	−	−
**Osmotic stress**	*opuA*	−	−	−	−	−	−	−	−
	*opuC*	+	+	+	+	+	+	+	+
	*opuBD*	−	−	−	−	−	−	−	−
	*opuCD*	+	+	+	+	+	+	+	+
	*opuCC*	+	+	+	+	+	+	+	+
	*gbuA*	−	−	−	−	−	−	−	−
	*gbuB*	−	−	−	−	−	−	−	−
	*gbuC*	−	−	−	−	−	−	−	−
**Bacteriocin**	*EnterolysinA* *(class lll bacteriocin)*	+	+	+	+	+	+	+	+

(+) gene detected in the genome based on Prokka annotation; (−) gene not detected.

## Data Availability

The original contributions presented in this study are included in the article/Supplementary Material. Further inquiries can be directed to the corresponding author(s).

## Supplementary Materials

The following supporting information can be downloaded at: https://www.mdpi.com/article/10.3390/ijms27135996/s1.
