# Supplementary material for: Comparative Genomic and Functional Characterization of Pediococcus acidilactici Isolated from Fermented Cacao with Anti-ESKAPE Activity
Source: Int J Mol Sci. 2026 Jul 3;27(13):5996. doi: 10.3390/ijms27135996 (PMC13362338; doi:10.3390/ijms27135996)
Supplement: Supplementary file 1 [file ijms-27-05996-s001.zip › ijms-4372334-supplementary.pdf]

**Supplementary Table**

**Supplementary Table S1:** LAB isolates from fermented cacao through Morphological, physiological, and Bruker Biotyper classification results

| **Source** | **Isolates** | **Cell shape** | **Gram staining** | **Catalase** | **Species** |
|---|---|---|---|---|---|
| Fermented cacao | CR03 | Cocci | positive | negative | *Pediococcus acidilactici* |
| | CR04 | Cocci | positive | negative | *Pediococcus acidilactici* |
| | CR05 | Cocci | positive | negative | *Pediococcus acidilactici* |
| | CR06 | Cocci | positive | negative | *Pediococcus acidilactici* |
| | CR07 | Cocci | positive | negative | *Pediococcus acidilactici* |
| | CR08 | Cocci | positive | negative | *Pediococcus acidilactici* |
| | CR11 | Cocci | positive | negative | *Pediococcus acidilactici* |
| | CR12 | Cocci | positive | negative | *Pediococcus acidilactici* |

**Supplementary Table S2:** Antibiotic susceptibility of *Pediococcus acidilactici*

| **Isolates** | Ampicillin (10 ug) | Vancomycin (30 ug) | Gentamicin (10 ug) | Erytomycin (15 ug) | Clindamycin (2 ug) | Tetracycline (30 ug) | Kanamycin (30 ug) | Chloramphe-nicol (30 ug) | Streptomy-cin (10 ug) |
|---|---|---|---|---|---|---|---|---|---|
| CR03 | R | R | S | R | R | S | R | S | R |
| CR04 | R | R | S | R | R | S | R | S | R |
| CR05 | R | R | S | R | R | S | R | S | R |
| CR06 | R | R | S | R | R | S | R | S | R |
| CR07 | R | R | S | R | R | S | R | S | R |
| CR08 | R | R | S | R | R | S | R | S | R |
| CR11 | R | R | S | R | R | S | R | S | R |
| CR12 | R | R | S | R | R | S | R | S | R |

*R* : resistant, *I* : intermediate, *S* : susceptible

## Supplementary Figure

**CR03**

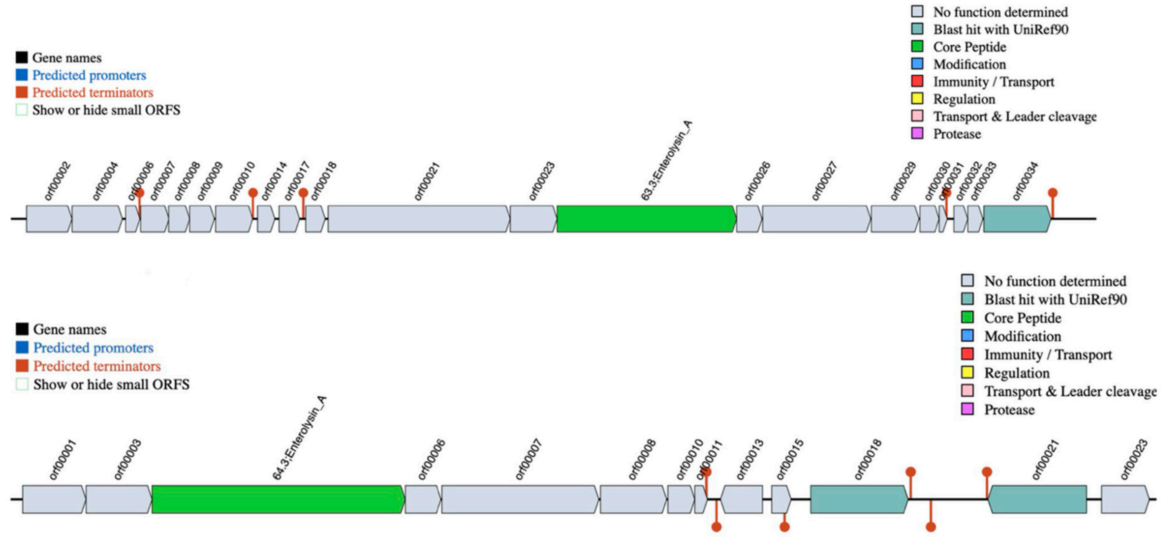


**CR04**

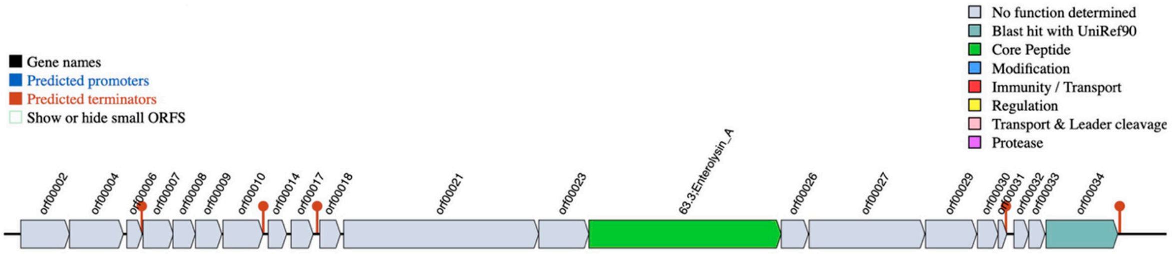

**CR05**

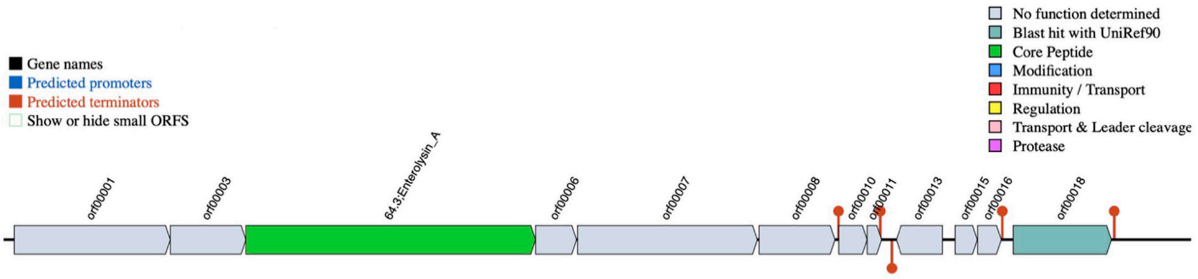


**CR06**

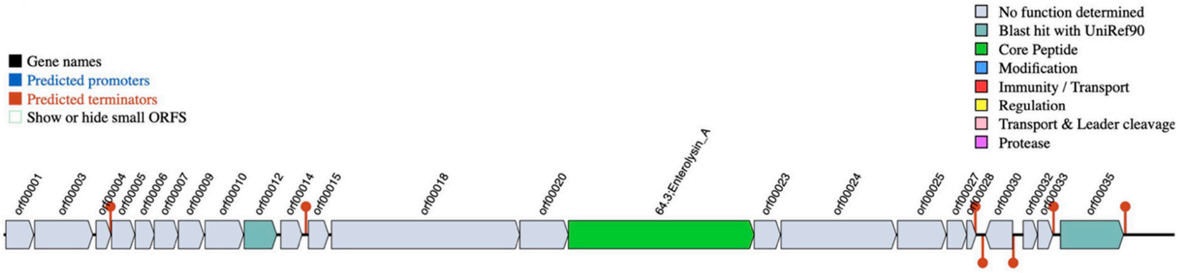


**CR07**

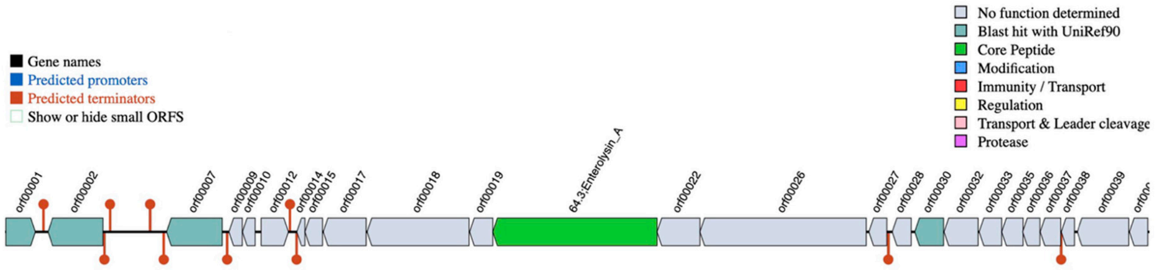


**CR08**

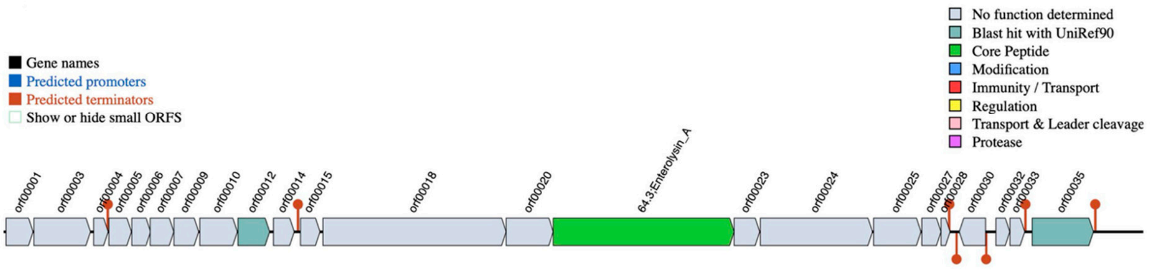

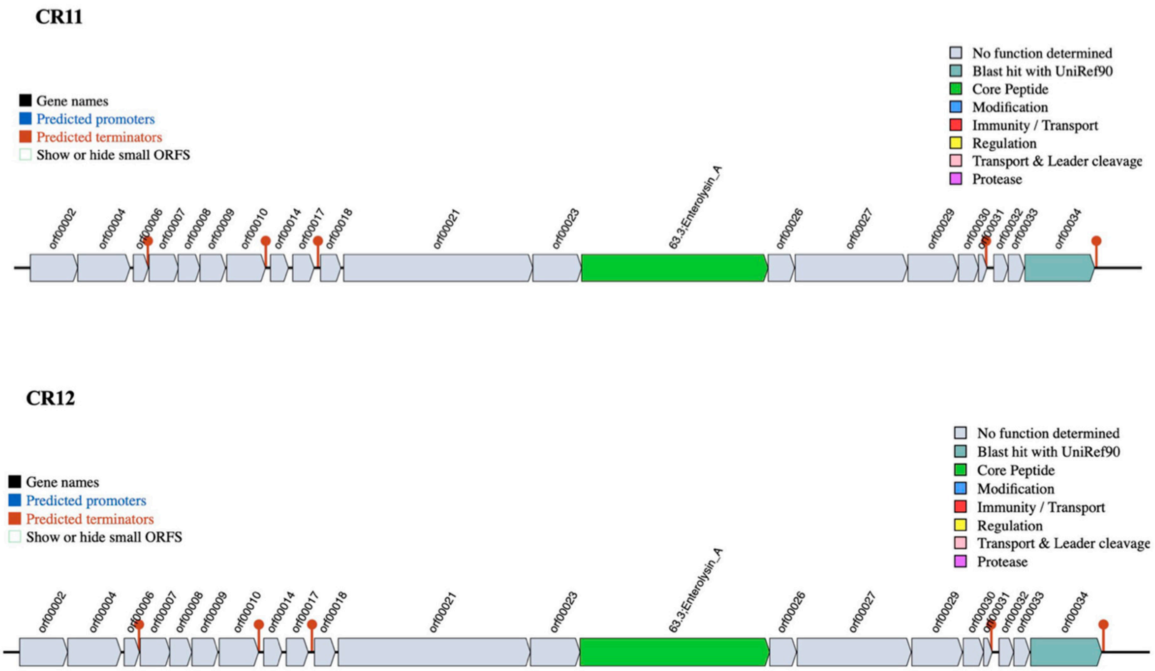


**Supplementary Figure S1:** Organization of predicted bacteriocin gene clusters in selected isolates based on BAGEL4 analysis. The clusters comprise genes encoding core peptides, including *enterolysin A*, along with accessory genes involved in modification, transport, immunity, and regulation. The conserved arrangement of these genes across isolates indicates a shared bacteriocin biosynthetic system and potential antimicrobial function.
